# Unveiling the Enigma of Statin-Induced Necrotizing Autoimmune Myopathy: A Comprehensive Case Analysis and Pathogenic Insights

**DOI:** 10.7759/cureus.69751

**Published:** 2024-09-19

**Authors:** Hemalatha Bhoompally, Praneeth Ulavala, Abhishek Vadher, Hari Chandana Kalangi, Swati Baraiya

**Affiliations:** 1 Intensive Care, Yashoda Hospitals, Secunderabad, IND; 2 General Internal Medicine, Narayana Medical College and Hospital, Nellore, IND; 3 Cardiology, Hofstra North Shore-LIJ School of Medicine, North Shore University Hospital, New York, USA; 4 General Medicine, Kamineni Academy of Medical Scieneces and Research Centre, Hyderabad, IND; 5 Family Medicine, Bombay Hospital and Medical Research Center, Mumbai, IND

**Keywords:** hmg-coa reductase antibodies, myopathy, neuromuscular weakness, statin, statin-induced necrotizing autoimmune myositis

## Abstract

Statins are widely prescribed to patients for their efficiency in cholesterol management. However, they carry the risk of inducing a relatively rare, serious, and potentially life-threatening condition known as statin-induced necrotizing autoimmune myopathy (SINAM). This case report provides an in-depth analysis of SINAM, focusing on its epidemiology, clinical presentation, and the challenges involved in accurate diagnosis. In addition, we review evolving theories addressing its pathogenesis and current therapeutic approaches. This report identifies the need for early diagnosis and intervention and recommends a multidisciplinary approach to optimize patient outcomes. This article also highlights the need for further research studies to enhance our understanding of SINAM and its management.

## Introduction

Extensive use of statins has undoubtedly changed the face of cholesterol management, reducing the burden of cardiovascular disease immensely. However, statins are associated with muscle-related side effects ranging from mild myalgia to severe and potentially fatal rhabdomyolysis. Somewhere in this spectrum lies SINAM (statin-induced necrotizing autoimmune myopathy), which is an extremely rare entity that requires meticulous attention for its devastating potential. SINAM is characterized by acute onset and progressive muscle weakness of the proximal type, increased creatine kinase (CK), and the presence of specific autoantibodies against HMG-CoA reductase. It requires timely and manifold strategies for preserving muscle functions and preventing long-term sequelae [1].

Epidemiology and risk factors

Although statin therapy is vital, there are still concerns about muscle-related side effects, most significantly myalgia, aching in the muscles, estimated to occur in 5-10% of subjects, and rhabdomyolysis in 0.1-0.2 per 100,000 person-years. SINAM is estimated to occur in two to three per 100,000 individuals taking statins [2-4]. There appears to be a predisposition in older adults, particularly females, although the specific risk factors are not well understood. Further exploration may be required for associations with higher statin doses, genetic predisposition, and coexisting autoimmune conditions [5].

Clinical features

Typical clinical presentation of SINAM usually includes acute, progressive muscle weakness of proximal distribution, more significant in the thighs and hip areas. Other complaints include difficulty climbing stairs, getting up from a sitting position, and walking. Myalgia, fatigue, and dysphagia may accompany the weakness, with a very prominent picture of functional impairment [4,6]. It is worth noting that early symptoms might be relatively mild and easily attributed to other causes; therefore, a high index of suspicion is required for its diagnosis.

Diagnosis

Diagnosis of SINAM requires a holistic approach because it is an unusual condition with variable presentations. The process should be initiated with a good history detailing the duration and pattern of weakness, use of statins, and past medical history. Physical examination focuses on assessing muscular strength and identifying weak regions [6].

Laboratory tests are significant, as CK levels are often elevated, typically more than 10 times the upper limit of normal [7]. Along with inflammatory markers and thyroid function, autoantibody panels are screened for, including the elusive, characteristic HMG-CoA reductase antibodies [4].

Imaging modalities such as magnetic resonance imaging (MRI) can be very useful in visualizing edema and inflammation of affected muscles and are essential for distinguishing from other myopathies. Electromyography (EMG) typically reveals fibrillations, positive sharp waves, and abnormal motor unit action potentials (MUAP), thus supporting a diagnosis of myopathic process [4,6,8].

This case report details the clinical course of a patient diagnosed with SINAM, presenting a unique challenge due to the absence of detectable HMG-CoA reductase antibodies.

## Case presentation

This is a case report of a 73-year-old obese lady who had a long history of diabetes mellitus, hypertension, and dyslipidemia. She was prescribed rosuvastatin 40 mg daily for several years. She was admitted with a three-day history of frequent loose stools, epigastric pain, and generalized body aches and weakness in all four limbs. She developed paraparesis with a muscle power of 2/5 in both lower limbs, more so in the proximal muscles, on clinical examination. The patient's muscle tone was normal with sluggish deep tendon reflexes and mute plantar reflexes.

**Figure 1:**
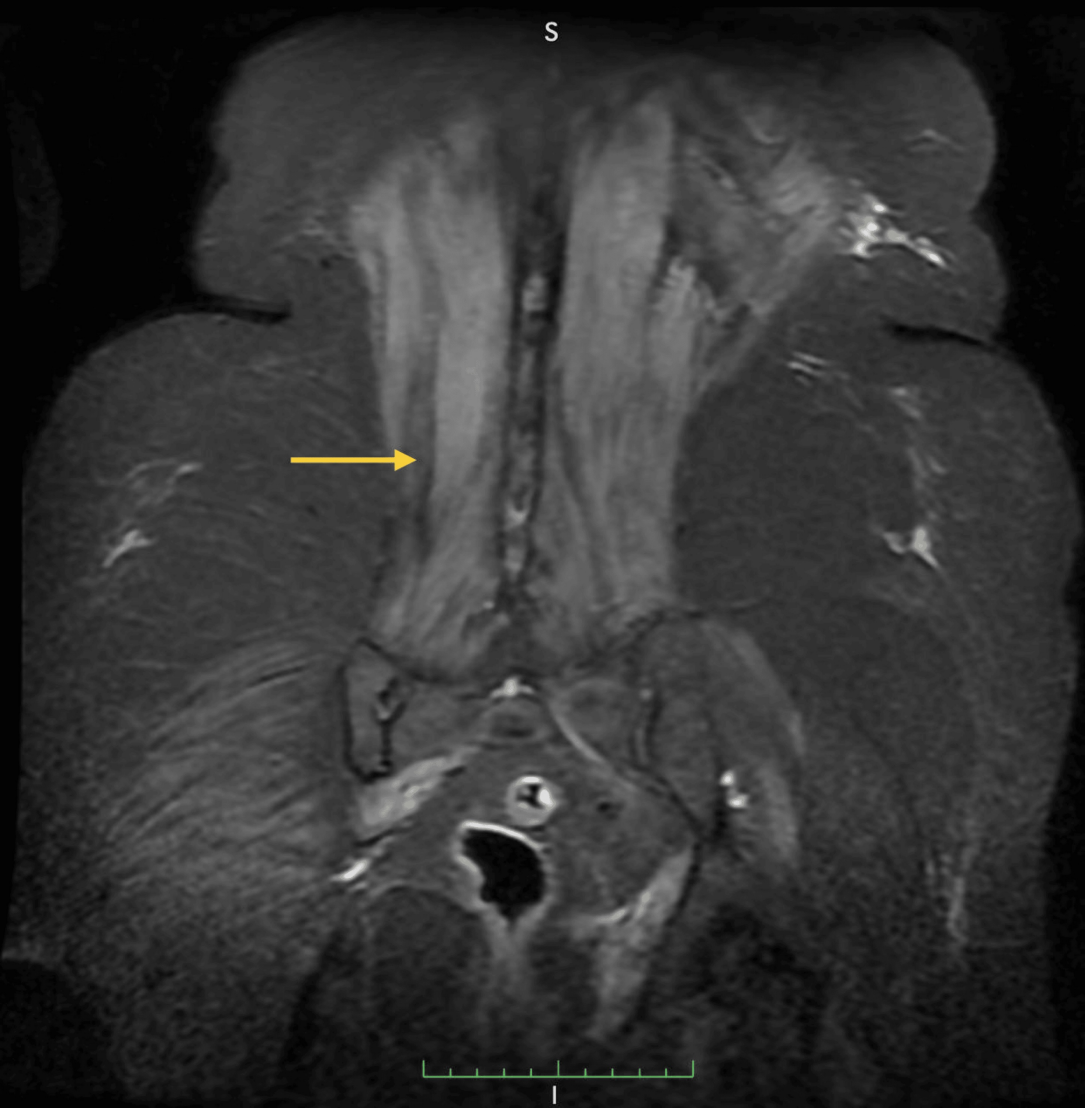
MRI of the spine showing myoedema in paraspinal, piriformis, and obturator muscles The yellow arrow in the figure points toward the myoedema in the paraspinal muscles MRI, magnetic resonance imaging

Initial investigations, including nerve conduction studies (NCS) and routine laboratory blood tests, did not explain the cause of this neuromuscular weakness. Further evaluation with EMG revealed spontaneous activity, including fibrillations, positive sharp waves, and occasional myotonic discharges. MUAP were increased in duration, with a reduced interference pattern consistent with a myogenic pattern. Her serum CK levels were 1000 U/L, which is about 10-fold higher than normal reference values (30-145 U/L). She also had acute kidney injury and hepatopathy. MRI imaging revealed myoedema in the paraspinal, piriformis, and obturator muscles, as shown by the yellow arrow in Figure 1. Autoimmune and metabolic workups were also negative, including anti-nuclear antibodies (ANA) profile, myositis profile, HMG-CoA reductase antibodies, urine porphobilinogen, and myoglobin.

The diagnosis based on these findings was SINAM. The patient was treated with intravenous pulse steroid therapy to good effect, showing a significant clinical improvement.

## Discussion

While significant improvements in understanding have been made, the exact mechanisms of SINAM are still poorly understood. Although most cases suggest an immune-mediated attack against muscle tissue based on positive results for HMG-CoA reductase autoantibodies, a subset of these patients present with negative serology and alternative ways of induction are being explored [9]. Evolving research investigates roles for T-cell cytotoxicity, cytokine dysregulation, and possible genetic susceptibility to provide more complete insights into SINAM pathogenesis [10].

The differentiation of SINAM from other myopathies is frequently difficult. One needs to consider the clinical presentation, laboratory findings, imaging, and results of EMG [11]. Also, a detailed drug history and other possible causes must be solicited.

The early withdrawal of statins, along with immunosuppressive treatment, is essential for the prevention of further muscle injury and subsequent long-term disability. Still, persistent muscular injury and residual functional impairment could be seen [12]. This again brings home the point about early recognition and aggressive management. Regular follow-up and intervention are necessary to optimize recovery and improve overall quality of life.

Our patient's presentation highlights the many complexities in diagnosing and managing SINAM, especially those without the characteristic HMG-CoA reductase autoantibodies. Without these characteristic diagnostic markers, even if all the other criteria are met, the search for alternative pathogenetic mechanisms is compelling and leads to the following discussion points.

Early recognition and intervention

It is crucial to acknowledge SINAM even with non-typical serology. The encouragement of statin withdrawal and early aggressive immunosuppressive therapy might have contributed to patient recovery [2,13]. This case report illustrates the need for vigilance and inclusion of SINAM in the differential diagnosis of similar symptoms, even in antibody-negative individuals.

Delving into alternative pathogenic mechanisms

The absence of HMG-CoA reductase antibodies requires us to explore outside the range of our current understanding of immune reactions triggered by these autoantibodies. There are other possible routes to consider, like investigating the role of T-cell activation and direct muscle fiber damage in antibody-negative cases, exploring the involvement of specific pro-inflammatory cytokines in triggering and sustaining muscle inflammation and also assessing potential genetic predispositions that might interact with statin exposure and contribute to SINAM development, even in the lack of these specific autoantibodies [6,8,14,15].

Refining diagnostic tools

Additional biomarkers with better sensitivity and specificity should be looked for improved early detection and more accurate diagnosis, particularly in antibody-negative cases [10]. Non-invasive methods like high-end blood tests or imaging techniques should be developed to reduce patient discomfort, and hence, intervention could happen at a very early stage [2]. Improvements in muscle biopsy methods and examination should be harnessed to gain further insights into the mechanisms behind diseases and potential targets of treatment [13].

Personalized treatment strategies

Exploring how pharmacogenomic testing influences individual treatment decisions, allowing for patients' susceptibility predictions based on their genetic profile, is essential to creating a customized treatment plan [16]. Assess the efficacy of highly targeted immunomodulatory treatments that may be more likely to optimize treatment outcomes and decrease side effects for the specific underlying causes of diseases.

Customized rehabilitation may also be considered for each patient based on their muscle involvement and functional limits. This will help in patients' recovery and improve the general level of health [17].

Preventative measures exploration

The search for genetic or phenotypic markers predictive of susceptibility to developing SINAM aims to facilitate appropriate early intervention and risk stratification [11,17]. To investigate pre-emptive interventions in high-risk subjects using low-dose immunosuppressive therapy or alternate lipid-lowering agents to prevent developing SINAM [2,10]. Clearly stating the criteria for statin monitoring and guidelines for cessation in patients with early signs or symptoms suspicious of SINAM to minimize possible complications [2,18].

Improve patient education and awareness

Educational materials should be prepared for patients and health professionals to spread awareness about SINAM, its symptoms, and the need for early consultation. Free and open communication should be promoted between patients and health professionals about their concerns regarding the muscle-related side effects of statin therapy. Another vital aspect is encouraging patient advocacy groups. This along with research initiatives in SINAM and its long-term complications should also be undertaken to embolden the patients further and take more research initiatives.

## Conclusions

SINAM is a very rare, potentially life-threatening complication. The latency of its presentation after statin use is variable, and early recognition is important to preserve muscle function and optimize outcomes. This case illustrates the critical role of early diagnosis and aggressive immunosuppressive therapy in preventing extensive muscle damage and enhancing strength recovery in HMG-CoA reductase antibody-negative SINAM.

Our case, however, also reminds us of the limitations in our current understanding of SINAM. Indeed, the absence of HMG-CoA reductase antibodies in the presence of fulfilling other diagnostic criteria calls for further investigation into alternative pathologic mechanisms beyond the described theory of "tolerance caused by up-regulation of HMG-CoA reductase" secondary to statin exposure.

Provided that there is an active involvement in these research and awareness efforts, this can lead us to a time when SINAM is not only effectively diagnosed and managed but also prevented. All this can significantly enhance the quality of life of the affected individuals with this complication and ensure that the best possible care is offered to them.
